# Zeolitic Imidazolate Framework-8 Nanoparticles Exhibit More Severe Toxicity to the Embryo/Larvae of Zebrafish (*Danio rerio*) When Co-Exposed with Cetylpyridinium Chloride

**DOI:** 10.3390/antiox11050945

**Published:** 2022-05-11

**Authors:** Xuchun Qiu, Lei Liu, Wei Xu, Chen Chen, Ming Li, Yanhong Shi, Xiangyang Wu, Kun Chen, Chongchen Wang

**Affiliations:** 1 Institute of Environmental Health and Ecological Security, School of the Environment and Safety Engineering, Jiangsu University, Zhenjiang 212013, China; xuchunqiu@ujs.edu.cn (X.Q.); llleiliu@163.com (L.L.); f15751772606@163.com (W.X.); chenchen9688@yeah.net (C.C.); liming@ujs.edu.cn (M.L.); shiyanhong2003@126.com (Y.S.); wuxy@ujs.edu.cn (X.W.); 2 Jiangsu Collaborative Innovation Center of Technology and Material of Water Treatment, Suzhou University of Science and Technology, Suzhou 215009, China; 3 Beijing Key Laboratory of Functional Materials for Building Structure and Environment Remediation, School of Environment and Energy Engineering, Beijing University of Civil Engineering and Architecture, Beijing 100044, China

**Keywords:** behavioral responses, cetylpyridinium chloride, combined effects, oxidative stress, zeolite imidazolate framework nanoparticles, zebrafish

## Abstract

The combined application of nanoparticles and surfactants has attracted tremendous attention in basic research and industry. However, knowledge of their combined toxicity remains scarce. In this study, we exposed zebrafish embryos to cetylpyridinium chloride (CPC, a cationic surfactant, at 0 and 20 μg/L), zeolitic imidazolate framework nanoparticles (ZIF-NPs, at 0, 30, and 60 mg/L), and their mixtures until 120 h post-fertilization (hpf). Within the used concentration range, both single and combined exposures exhibited limited effects on the survival and hatching of zebrafish. However, the combined exposure of ZIF-NPs and CPC caused more severe effects on the heart rate at both 48 and 72 hpf. The combined exposure also induced significant hyperactivity (i.e., increasing the average swimming velocity) and oxidative stress in zebrafish larvae (at 120 hpf), although all single exposure treatments exhibited limited impacts. Furthermore, the level of reactive oxygen species (or malondialdehyde) exhibited a significantly positive correlation with the heart rate (or average swimming velocity) of zebrafish, suggesting that oxidative stress plays a role in mediating the combined toxicity of CPC and ZIF-NPs to zebrafish. Our findings suggest that the interaction of CPC and ZIF-NPs should not be ignored when assessing the potential risks of their mixtures.

## 1. Introduction

In recent years, the combined application of nanoparticles (NPs) and surfactants have attracted tremendous attention in basic research and industry [1,2,3,4]. For example, the application of surfactant-coated nanoparticles demonstrated that surfactants could further enhance the functions of the NPs, and their synergistic effects have created many useful new technologies in nanomedicine and food nanotechnology [1]. The nanoparticle–surfactant nanofluid, composed of NPs and surfactants, can play the oil displacement mechanism of surfactant and nanofluid simultaneously and has a great application prospect in enhanced oil recovery [3,4]. Therefore, understanding the combined impacts of NPs and surfactants is essential to avoid negative impacts and design safer materials [1,2].

Zeolitic imidazolate framework nanoparticles (ZIF-NPs) belong to metal-organic framework (MOFs) materials, which are considered to be ideal candidates for various applications such as energy recovery and storage, catalysis, sensors, and environmental remediation [5,6,7]. Furthermore, the ZIF-NPs exhibited a high potential as antibacterial platforms against oral diseases [7,8]. On the other hand, cetylpyridinium chloride (CPC), a quaternary ammonium cationic surfactant, has also been widely used as an antibacterial ingredient in pharmaceutical and personal care products, especially in oral products [9]. In addition to the similar therapeutic application, both ZIF-NPs and CPC have a high potential to enhance oil recovery in the petroleum industry [4,5,6]. However, whether and how their mixtures induce health or environmental impacts remain unclear.

Previous studies have shown that single exposure to ZIF-NPs or CPC might induce various physiological defects in model organisms, many associated with oxidative stress. For example, Johari et al. [10] reported that exposure of human embryonic kidney cells to ZIF-8 NPs could increase the intracellular reactive oxygen species (ROS) production and active apoptosis pathway, and Chen et al. [11] found that exposure to ZIF-8 NPs leads to high ROS level and cellular inflammation in human HepG2 cells. Using rat thymic lymphocytes, Imai et al. [12] found that CPC exerted cytotoxic effects under oxidative stress conditions by increasing intracellular Zn^2+^ concentration and decreasing the cellular content of nonprotein thiols. In recent years, Bhattacharya and colleagues found that sublethal CPC exposure could adversely affect the antioxidant enzymes in aquatic worms and common carp [13,14,15]. Our previous study also suggested that CPC at 400 µg/L significantly elevated the reactive oxygen species (ROS), superoxide dismutase (SOD), and glutathione (GSH) levels in zebrafish larvae [16]. However, knowledge of the combined toxicity of ZIF-NPs and CPC and related mechanisms remains scarce.

Zebrafish (*Danio rerio*) is a model organism that has been widely used for assessing the toxicological effects of chemicals [17,18,19] and NPs [20,21,22]. Furthermore, the high degree of genetic similarity with humans supports the notion that zebrafish can be used as a complementary research tool for human health risk assessment [23]. Therefore, we conducted a combined exposure of zebrafish embryos to CPC, ZIF-NPs, and their mixtures, and investigated their survival, development, hatching, oxidative stress, and behavioral responses within early life stages. This study aimed to investigate the combined toxicity of CPC and ZIF-NPs to the early life stages of zebrafish and the contribution of their interaction to the final impact.

## 2. Materials and Methods

### 2.1. Chemical

Cetylpyridinium Chloride (CAS No. 123-03-5) was purchased from the Shanghai Yien Chemical Technology Co., Ltd. (Shanghai, China). Other reagents at analytical grade were purchased from the Sinopharm Chemical Reagent Co., Ltd. (Shanghai, China). The ZnO and 2-methylimidazole were commercially available from J&K Scientific LLC (San Jose, CA, USA). The zeolitic imidazolate framework-8 nanoparticles (ZIF-NPs) were produced via the mechanochemical reaction between ZnO and 2-methylimidazole with a molar ratio of 1:2 [24]. The ZIF-NPs were characterized by scanning electron microscopy (SEM; SU8020, Hitachi Limited, Tokyo, Japan), which showed their shapes with 100–400 nm particle sizes (see Supplementary Figure S1). The enzyme-linked immunosorbent assay (ELISA) kits for assaying the reactive oxygen species (ROS), total superoxide dismutase (T-SOD), glutathione (GSH), catalase (CAT), and malondialdehyde (MDA) were purchased from the Bomei Biotechnology Co., Ltd. (Hefei, Anhui, China).

### 2.2. Test Organisms

Adult zebrafish (AB Strain) used for spawning was maintained in our laboratory for over four months. Male and female zebrafish were separately placed in two 16-L glass aquariums (28 cm diameter and 30 cm height) containing 12 L dechlorinated tap water (conductivity at 0.50–0.53 mS/cm) at 27 ± 1 °C, and cultured under light: darkness = 14:10 h cycles. Zebrafish were fed with newly hatched *Artemia nauplii* twice a day, and half of the water was renewed every two days.

A total of 50 healthy zebrafish (30 females and 20 males) were used for spawning. First, three females and two males were placed in a spawning box (Aqua Schwarz GmbH, Goettingen, Germany) and temporarily separated by a baffle in the afternoon before the exposure test. On the following day (i.e., the day for the exposure test), the baffles of all spawning boxes were removed just after the light was turned on at 8:30 am. After 15 min, the embryos were transferred to a Petri-dish containing E3 medium (5 mM NaCl, 0.17 mM KCl, 0.33 mM CaCl_2_, 0.33 mM MgSO_4_ in dH_2_O) [25] and washed three times using the medium. The embryos were observed under a stereomicroscope (SZX16, Olympus, Tokyo, Japan), and only healthy embryos (i.e., fertilized, starting division, and without any deformity) were transferred to clean Petri-dishes for exposure test.

### 2.3. Exposure Experiment

Our previous studies reported that the LC_50_ value of CPC and ZIF-NPs for early the early life stages of zebrafish were 176 μg/L (120 h-LC_50_) and 643 mg/L (96 h-LC_50_), respectively [16,26]. In the present study, the maximum exposure concentrations of CPC and ZIF-NPs were set to be approximately 1/10 of their respective LC_50_ values. The test solutions were prepared by adding calculated amounts of ZIF-NPs stock solution (6000 mg/L in deionized water) and CPC stock solution (60 mg/L in deionized water) into E3 medium. The following experimental groups were designed: (1) Control: ZIF-NPs = 0 mg/L, CPC = 0 µg/L; (2) NPs30: ZIF-NPs = 30 mg/L, CPC = 0 µg/L; (3) NPs60: ZIF-NPs = 60 mg/L, CPC = 0 µg/L; (4) CPC20: ZIF-NPs = 0 mg/L, CPC = 20 µg/L; (5) Mix3020: ZIF-NPs = 30 mg/L, CPC = 20 µg/L; (6) Mix6020: ZIF-NPs = 60 mg/L, CPC = 20 µg/L.

In each experimental group, 150 healthy embryos were randomly introduced to three Petri-dishes (50 embryos per dish, *n* = 3) containing 20 mL of corresponding test solution. The Petri-dish was placed in an incubator (14L:10D) at 28 °C until 120 h post-fertilization (hpf), and the test solution was renewed every 24 h. The mortality, hatching time, and abnormal development were confirmed at the 1, 2, 4, 8, 24, 48, 72, 96, and 120 hpf, and the dead embryos were removed in time. At the 48 and 72 hpf, the heart rate of 6 random individuals in each Petri-dish was manually counted, following the method described by Qiu et al. [16].

### 2.4. Light-Dark Locomotion Test of Newly Hatched Larval

At the 120 hpf, eight larvae were randomly selected from each Petri-dish for the light–dark locomotion test. The larvae were carefully transferred to 24-well plates with a single larva per well (diameter = 15 mm, including 800 μL E3 medium). After a 10-min acclimation period in the dark condition, the behavioral response of larvae to alternating cycles of light and dark (i.e., 10 min dark-10 min light-10 min dark) was recorded by a DanioVision System (Noldus, Wageningen, Netherlands). Subsequently, the average swimming velocity (ASV, mm/s) was analyzed using EthoVision XT software (Vision 11.5; Noldus, Wageningen, Netherlands).

### 2.5. Biochemical Assays

At the 120 hpf, ROS, T-SOD, GSH, CAT, and MDA levels in zebrafish larval were determined. All survival larvae in each Petri-dish were pooled as a sample for those assays. The homogenate and supernatant were prepared following the method described by Qiu et al. [16]. The supernatant of each sample was used for biochemical assays, following the manufacturer’s instructions for the corresponding ELISA Kit. Those ELISA Kits use the Sandwich Assay Method, using the TMB (3,3′,5,5′-tetramethylbenzidine) as the chromogenic substrate for horseradish peroxidase to generate the signal. Thus, the optical density (OD) was measured by a Microplate Spectrophotometer (Synergy H4, BioTek, Winooski, VT, USA) at a 450-nm wavelength. The units were normalized by the total protein amount in each sample (measured by a BCA kit) and were expressed as per mg-protein (/mg-protein).

### 2.6. Statistical Analysis

The combined effects of CPC and ZIF-NPs on the biochemical and behavioral parameters were analyzed by a generalized linear model (GzLM) that was performed using a Gaussian distribution and identity link function, considering the concentrations of CPC and ZIF-NPs as categorical variables. For the subsequent pairwise comparison, the experimental data were checked for assumptions of homogeneity of variance using Levene’s test. Because the ROS level could not satisfy the assumptions of homogeneity of variance, the nonparametric Mann–Whitney U-test was used to test for differences between treatments. The statistical significance of correlations between oxidative stress-related biomarkers and biological or behavioral parameters was tested by Spearman’s correlation analysis. All statistical analyses were performed using SPSS 16.0 (SPSS Inc., Chicago, IL, USA).

## 3. Results

### 3.1. Effects on Mortality and Hatching

**Figure 1:**
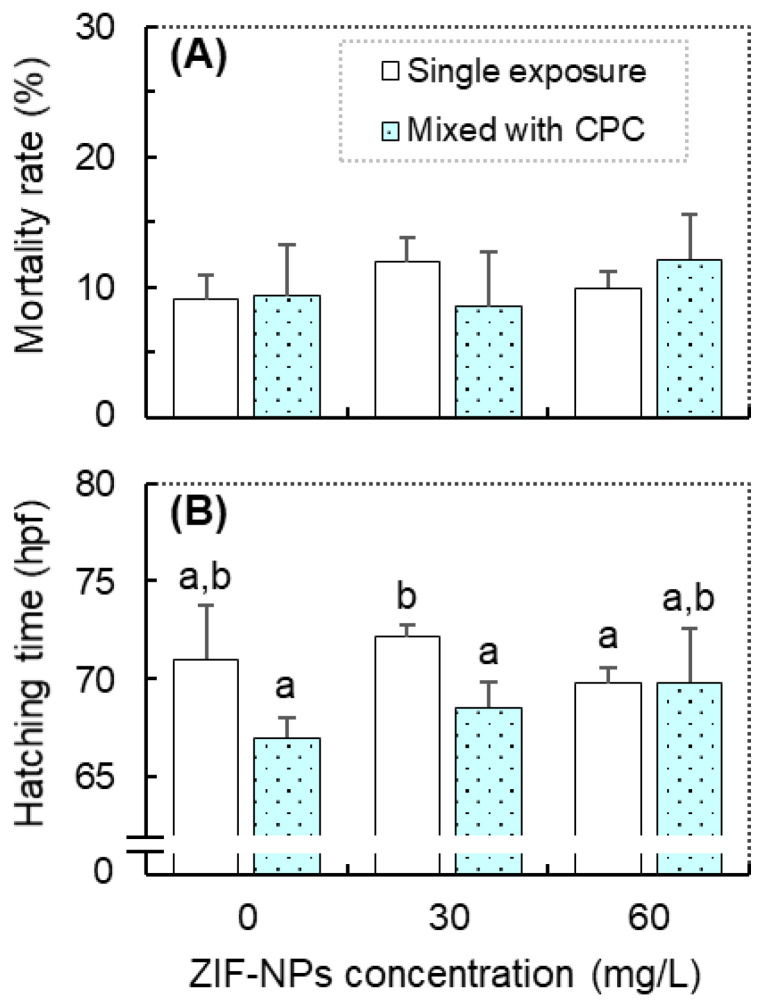
The mortality rate (**A**) and average hatching time (**B**) of zebrafish (*Danio rerio*) in the combined exposure groups of cetylpyridinium chloride (CPC, 0 and 20 μg/L) and ZIF-8 nanoparticle (ZIF-NPs; 0, 30, and 60 mg/L). Data are means ± SE (*n* = 3), and values that do not share a common letter are significantly different at *p* < 0.05.

As shown in Figure 1, combined exposure to ZIF-NPs and CPC exhibited limited effects on the survival and hatching time. Regardless of the exposure combinations, no significant difference in the mortality rate of zebrafish was observed over the different treatments (Figure 1A). The GzLM analysis also revealed no statistical significance for the effects of CPC, ZIF-NPs, and their interaction on the mortality rate of fish (Table 1). Compared with the control, there was no significant difference in the average hatching time of embryos in the exposure groups (Figure 1B). However, combined exposure to CPC and ZIF-NPs (30 mg/L) significantly reduced the average hatching time of embryos compared to the single ZIF-NPs (30 mg/L) exposure group. In addition, the GzLM analysis revealed that CPC exhibited a significant main effect on the hatching time of embryos (Table 1).

### 3.2. Effects on the Heart Rate

**Figure 2:**
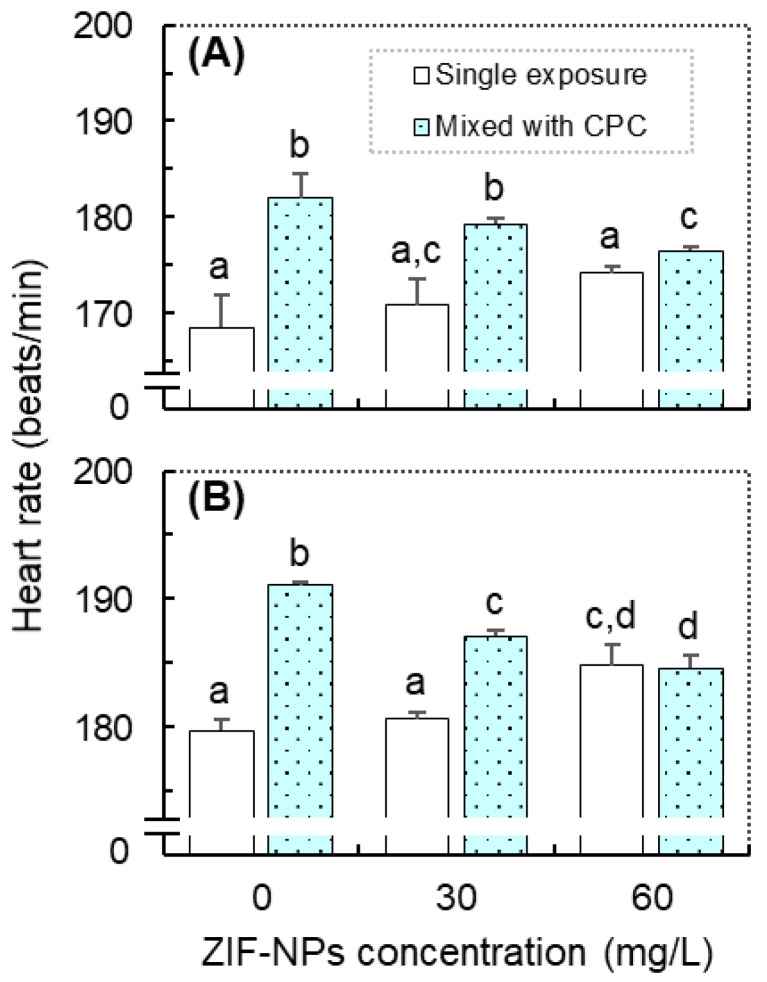
The heart rates of zebrafish (*Danio rerio*) in the combined exposure groups of cetylpyridinium chloride (CPC; 0 and 20 μg/L) and ZIF-8 nanoparticle (ZIF-NPs; 0, 30, and 60 mg/L). Data are means ± SE (*n* = 3), which were measured at 48 (**A**) and 72 (**B**) hours post-fertilization (hpf). Values that do not share a common letter are significantly different at *p* < 0.05.

The heart rates of zebrafish at 48 hpf (embryos) and 72 hpf (larvae) are shown in Figure 2. The GzLM analysis showed that CPC exhibited a significant main effect on the heart rate at both 48 and 72 hpf, ZIF-NPs exhibited a significant main effect on heart rate at 72 hpf, and the interaction exhibited significant effects on the heart rate at both 48 and 72 hpf. At 48 hpf, the single exposure to ZIF-NPs had no significant effect on the heart rate, but single exposure to CPC significantly increased the heart rates of embryos (vs. control, Figure 2A). Moreover, both combined exposures significantly increased the heart rates of embryos compared with the control and corresponding single ZIF-NPs exposure (Figure 2A). At 72 hpf, the single exposure to CPC (20 μg/L) or ZIF-NPs (60 mg/L) significantly increased the heart rate of larvae (vs. control, Figure 2B). Moreover, both combined exposures significantly increased the heart rate of zebrafish larvae (vs. control, Figure 2B).

### 3.3. Effects on the Behavioral Traits

**Figure 3:**
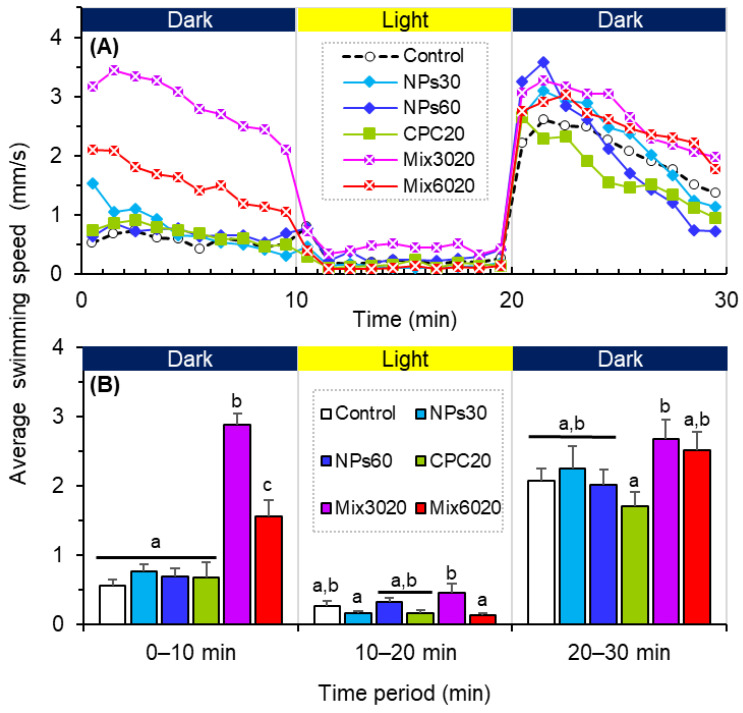
The average swimming velocity of larval zebrafish (*Danio rerio*) in the combined exposure groups of cetylpyridinium chloride (CPC; 0 and 20 μg/L) and ZIF-8 nanoparticle (ZIF-NPs; 0, 30, and 60 mg/L). (**A**) Mean value within each 1-min interval. (**B**) Means ± SE (*n* = 18) within each 10-min period, and values that do not share a common letter are significantly different at *p* < 0.05. Control: ZIF-NPs = 0 mg/L, CPC = 0 µg/L; NPs30: ZIF-NPs = 30 mg/L, CPC = 0 µg/L; NPs60: ZIF-NPs = 60 mg/L, CPC = 0 µg/L; CPC20: ZIF-NPs = 0 mg/L, CPC = 20 µg/L; Mix3020: ZIF-NPs = 30 mg/L, CPC = 20 µg/L; Mix 6020: ZIF-NPs = 60 mg/L, CPC = 20 µg/L.

Under the light and dark shifts, a single exposure to CPC or ZIF-NPs did not significantly change the average swimming velocity (ASV) of zebrafish larvae (vs. control); however, the mixed exposure significantly elevated their locomotor activity (i.e., hyperactivity), especially in the dark phases (Figure 3A). The results of the GzLM fit (Table 1) indicated that CPC and ZIF-NPs exhibited significant main effects on the ASV during the first dark period (i.e., 0–10 min), and their interaction exhibited significant effects on the ASV of larval zebrafish during the first dark period (i.e., 0–10 min) and the light period (i.e., 10–20 min). Compared with the control and single exposure groups, significantly higher ASV was observed in both combined exposures within the first 10-min dark period (Figure 3B). In the 10-min light period, the ASV of all zebrafish larvae became relatively low, and no significant difference was observed between each exposure group and the control group (Figure 3B). In the second 10-min dark period, the ASV of larval zebrafish also tended to increase in the combined exposure groups, and significant differences were observed between the single CPC exposure and the combined exposure to CPC and 30-mg/L ZIF-NPs (Figure 3B).

### 3.4. Responses in the Oxidative Stress-Related Biomarkers and Correlation Analysis

**Figure 4:**
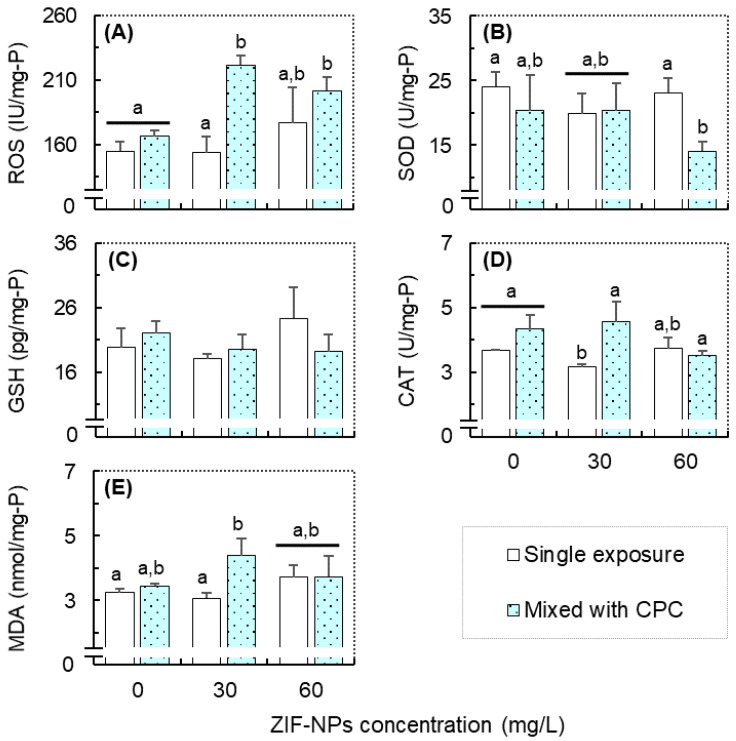
The levels of ROS (**A**), T-SOD (**B**), GSH (**C**), CAT (**D**), and MDA (**E**) in larval zebrafish in the combined exposure groups of cetylpyridinium chloride (CPC; 0 and 20 μg/L) and ZIF-8 nanoparticle (ZIF-NPs; 0, 30, and 60 mg/L). Data are mean ± SE (*n* = 3), and values that do not share a common letter are significantly different at *p* < 0.05.

Compared with the control, a single exposure to CPC or ZIF-NPs exhibited limited effects on the levels of oxidative stress-related biomarkers; however, their combined exposure tended to induce significant oxidative stress and modulate some antioxidant enzymes in zebrafish larvae (Figure 4). For the ROS level, significantly higher values were observed in both combined exposure groups compared with the control or the single exposure to CPC or 30-mg/L ZIF-NPs (Figure 4A). For the T-SOD level, a significantly lower value was only observed in the combined exposure to CPC and 60-mg/L ZIF-NPs, compared with the control or the single exposure to 60-mg/L ZIF-NPs (Figure 4B). For the GSH content, no significant difference was observed between the experimental groups (Figure 4C). For the CAT level, a significantly lower activity was only observed in the exposure group of 30-mg/L ZIF-NPs, compared with the single exposure to CPC or the mixture of CPC and 30-mg/L ZIF-NPs (Figure 4D). For the MDA level, a significantly higher value was observed in the combined exposure to CPC and 30-mg/L ZIF-NPs, compared with the control or the single exposure to 30-mg/L ZIF-NPs. The GzLM analysis predicated that CPC exhibited significant main effects on the levels of ROS, CAT, and MDA, ZIF-NPs exhibited a significant main effect on the level of ROS, and their interaction exhibited significant effects on the level of ROS, CAT, and MDA (Table 1).

The results of Spearman’s correlation analysis are shown in Table 2. The ROS and MDA levels exhibited significantly positive correlations with the heart rate of embryos (i.e., at 48 hpf) and larvae (i.e., at 72 hpf). The GSH concentration and CAT activity also exhibited significant positive correlations with the heart rate of larvae (i.e., at 72 hpf). For the behavioral parameters, the ROS level was significantly positively correlated with the ASV of larvae during the first (i.e., 0–10 min) and second (i.e., 20–30 min) dark periods; and the MDA level was significantly positively correlated with the ASV of larvae during the second dark period (i.e., 20–30 min).

## 4. Discussion

Within the concentration range used in this study, exposure to CPC, ZIF-NPs, and their mixtures exhibited limited effects on the survival and hatching of early life stages of zebrafish. Previous studies have reported that the LC_50_ value of CPC and ZIF-NPs for the early life stages of zebrafish were 176 μg/L (120 h-LC_50_) and 643 mg/L (96 h-LC_50_), respectively [16,26]. Those acute lethal concentrations were an order of magnitude higher than the exposure concentrations of CPC and ZIF-NPs, which may explain the limited lethal toxicity observed in this study. However, the combined exposure to CPC and ZIF-NPs could significantly affect the development, behavioral responses, and oxidative stress of zebrafish. Furthermore, the interaction between CPC and ZIF-NPs played significant roles in those sublethal effects, which should not be ignored when assessing the potential risks of their mixture.

Heart rate is often used as an important endpoint for assessing cardiac and developmental toxicity of chemicals to fish species [27,28,29,30,31]. In this study, significantly increased heart rate was observed in the single exposure to CPC at 20 μg/L or ZIF-NPs at 60 mg/L. Interestingly, previous studies have shown that exposure to CPC (40 and 400 μg/L) or ZIF-NPs (90 and 900 mg/L) could significantly reduce the heart rate in the early life stages of zebrafish [16,26]. These inconsistent results may be explained by the dose-specific biphasic effects of pollutants on aquatic species, which have been well documented by previous studies [27,28,29]. The increased or decreased heart rate of fish can be related to abnormal cardiac function induced by pollutants [27,28,29,30]. The increased heart rate may trigger significant cardiomyocyte death [29], whereas the reduced heart rate is related to the underdeveloped heart and pericardium or the high level of apoptotic heart cells [27].

Furthermore, the GzLM fit estimated that the interaction of CPC and ZIF-NPs significantly affected the heart rate at both 48 and 72 hpf. Similarly, previous studies also demonstrated that the interaction of chemicals and NPs might significantly affect their combined cardiotoxicity to zebrafish [32,33,34]. For example, Du et al. [32] found that combined exposure of ZnO-NPs and perfluorooctane sulfonate (PFOS) could cause a more severe effect on the heart rate of zebrafish embryos (48 hpf) compared with the control and single-treatments, and Saputra et al. [33] reported that co-treatment of CuO-NPs and carbofuran enhances cardiotoxicity in zebrafish embryos. Those previous reports and our findings highlight the necessity of further studies on the combined exposure to NPs and chemical pollutants [34].

The behavior is a comprehensive physiological response of animals to the environment, regarded as a sensitive endpoint or early warning signal of environmental pollution [35,36,37,38]. In this study, although a single exposure to CPC and ZIF-NPs did not significantly affect the locomotor activity of zebrafish, their combined exposures triggered significant hyperactivity, suggesting a synergistic effect. Hyperactivity is a common response of fish to pollutants [38], including CPC [16] and NPs [26]. In the early life stages of zebrafish, hyperactivity is associated with neural circuity components of their locomotor network [37]. Movement is essential for the health and survival of organisms as a key factor in foraging, social interaction, and defensive activities [35,36,37]. However, hyperactivity in fish may also attract a predator’s attention, leading to an increased predation risk [39]. In a natural environment, predation is one of the most important forces affecting the abundance and distribution of aquatic communities [39,40,41]. Thus, the hyperactivity resulting from their combined exposure may further affect the individual fate and even population dynamics.

Our results suggest that CPC and ZIF-NPs may also play a synergistic role in inducing oxidative stress in zebrafish larvae, as indicated by the notable increases in the ROS and MDA levels in the combined exposure groups and the significantly interactive effect predicated by the GzLM analysis. Numerous studies have demonstrated that oxidative stress is a common response in aquatic species to environmental pollutants, including NPs [20,21,22] and CPC [13,14,15,16]. Generally, organisms have evolved many enzymes (e.g., SOD, CAT, Glutathione S-Transferase, Glutathione Peroxidase, and Ascorbate Peroxidase) and antioxidants (e.g., GSH and Alpha-lipoic Acid) to scavenge excess ROS and protect cells from severe oxidative damage [42]. For example, exposure to CPC at 400 μg/L notably increased the ROS level in zebrafish larvae; at the same time, it also significantly increased the T-SOD activity and GSH content, finally resulting in a limited effect on the MDA (an indicator of oxidative stress-mediated lipid peroxidation) level [16]. In this study, however, combined exposure to CPC and 30-mg/L ZIF-NPs did not significantly increase the GSH content and CAT activity, which may further aggravate the oxidative damage of ROS accumulation. Indeed, a significantly higher MDA level of zebrafish larvae was observed in this combined exposure group compared with the control or the single exposure to 30-mg/L ZIF-NPs. Furthermore, ROS-induced lipid peroxidation plays a critical role in cell death [43], and many previous studies also proved that ROS accumulation induced by CPC or NPs exposure could activate apoptosis pathways in mammalian cell lines [10,11,12]. Thus, we inferred that similar phenomena might also occur in zebrafish larvae exposed to the mixture of CPC and ZIF-NPs. Nevertheless, more evidence (e.g., histochemical analysis, DNA fragmentation, and expression of related genes) is needed to confirm the above inference, which requires more future studies.

In contrast, combined exposure to CPC and 60-mg/L ZIF-NPs did not significantly increase the MDA level in zebrafish larvae; even this mixture significantly elevated their ROS level and decreased their T-SOD activity. A possible explanation is the dose-specific effects of ZIF-NPs on organisms, which have been well documented by recent studies [11,44,45]. Another possible explanation is that the interactions between components in mixtures exhibit complex effects on their joint toxicity, which is affected by many factors, such as the combination of a mixture, their dose level, dose ratio, and others [46,47]. For example, Du et al. [48] also found a significant induction of the ROS accompanied by the increased MDA contents in zebrafish embryos co-exposed to ZnO-NPs and PFOS, which exhibited synergistic effects. However, Ogunsuyi et al. [49] reported that the combined exposure to silver (Ag) and copper oxide (CuO) NPs exhibited antagonistic effects on the oxidative damage in the liver of catfish (*Clarias gariepinus*). Thus, the interaction of the components in a mixture may play very different roles in determining their combined toxicity, which highlights the importance of a systematic assessment of their toxicity mechanisms.

The results of the correlation analysis suggest that oxidative stress may be a mechanism to mediate the toxicity of combined exposure to CPC and ZIF-NPs to zebrafish. Previous studies have shown that ROS acts as signal molecules to mediate embryonic development processes in zebrafish [50], but excess ROS may lead to heart looping disorder during heart development [51]. Thus, the significantly positive correlations between heart rate and oxidative stress-related parameters indicate that oxidative stress may play a role in cardiac toxicity. It has also been reported that oxidative stress induced by environmental pollutants could trigger abnormal behaviors in the teleost with adverse ecological consequences [52,53,54]. Considering that ASV and ROS (or MDA) exhibited similar response trends and significant correlations, we inferred that combined exposure to CPC and ZIF-NPs may disrupt the behavior of zebrafish larvae by inducing oxidative stress to some extent.

## 5. Conclusions

In conclusion, our results demonstrated that combined exposure to CPC and ZIF-NPs might induce notable adverse effects on the development, behavior, and oxidative stress in early life stage zebrafish; even their single exposure generally exhibited limited impacts. Those impacts resulting from their combined exposure may further affect the individual fate and even population dynamics. Therefore, the interactive effects of CPC and ZIF-NPs should not be ignored when assessing their potential risks. Moreover, oxidative stress may play a role in mediating the combined toxicity of CPC and ZIF-NPs to zebrafish. However, little is known about the detailed mechanisms involved in the interactive effects of CPC and ZIF-NPs, which need to be investigated in future studies.

## Figures and Tables

**Table 1:** Summary of generalized linear model testing the statistical significance for the effect of cetylpyridinium chloride (CPC), ZIF-8 nanoparticles (ZIF-NPs), and their interaction (CPC × ZIF) on early life stages of zebrafish (*Danio rerio*) ^1.^

	Biological Parameters				Average Swimming Velocity			Oxidative Stress-Related Parameters				
	MOR	HAT	HB48	HB72	0–10 min	10–20 min	20–30 min	ROS	T-SOD	GSH	CAT	MDA
CPC (*df* = 1)	0.03	4.68 *	34.6 **	92.1 **	65.4 **	0.00	0.91	13.8 **	3.24	0.06	7.12 **	4.15 *
ZIF-NPs (*df* = 2)	0.64	1.09	0.06	11.8 **	58.9 **	2.07	5.75	17.7 **	2.06	2.40	2.64	4.13
CPC × ZIF (*df* = 2)	1.34	2.17	15.9 **	56.9 **	41.6 **	12.4 **	3.84	16.2 **	4.36	2.13	8.69 *	6.06 *

^1^ The asterisks following values indicate significant difference between treatment and control (* *p* < 0.05; ** *p* < 0.01). MOR: mortality rate; HAT: hatching time; HB48: heart rate of embryos at 48 hpf; HB72: heart rate of larvae at 72 hpf; ROS: reactive oxygen species; T-SOD: total superoxide dismutase; GSH: glutathione; CAT: catalase; MDA: malondialdehyde.

**Table 2:** Spearman’s correlation between the oxidative stress-related biomarkers and the heart rate or average swimming velocity (ASV) of zebrafish (*Danio rerio*) ^1^.

	Heart Rate		ASV Under the Light (L) and Dark (D) Shifts		
	48 hpf	72 hpf	0–10 min (D)	10–20 min (L)	20–30 min (D)
ROS	0.494 *	0.540 *	0.507 *	−0.063	0.513 *
T-SOD	−0.282	−0.126	−0.453	0.193	0.001
GSH	0.361	0.562 *	−0.119	−0.092	−0.007
CAT	0.441	0.663 **	0.073	0.108	0.127
MDA	0.485 *	0.558 *	0.411	0.417	0.485 *

^1^ Spearman’s correlation coefficient are listed, and the asterisks following indicate significant difference (* *p* < 0.05; ** *p* < 0.01).

## Data Availability

Data is contained within the article or Supplementary Materials.

## Supplementary Materials

The following supporting information can be downloaded at: https://www.mdpi.com/article/10.3390/antiox11050945/s1. Figure S1: Scanning electron microscopy (SEM) of the zeolitic imidazolate framework-8 nanoparticles.
