# Supplementary material for: Zeolitic Imidazolate Framework-8 Nanoparticles Exhibit More Severe Toxicity to the Embryo/Larvae of Zebrafish (Danio rerio) When Co-Exposed with Cetylpyridinium Chloride
Source: Antioxidants (Basel). 2022 May 11;11(5):945. doi: 10.3390/antiox11050945 (PMC9138101; doi:10.3390/antiox11050945)
Supplement: Supplementary file 1 [file antioxidants-11-00945-s001.zip › antioxidants-1693462-supplementary.pdf]

Supplementary material for

Zeolitic Imidazolate Framework-8 Nanoparticles Exhibit More Severe Toxicity to the Embryo/Larvae of Zebrafish (*Danio rerio*) When Co-Exposed with Cetylpyridinium Chloride

Xuchun Qiu [1,2], Lei Liu [1], Wei Xu [1], Chen Chen [1], Ming Li [1], Yanhong Shi [1], Xiangyang Wu [1] , Kun Chen [1,*] and Chong-Chen Wang [3,*]

[1] Institute of Environmental Health and Ecological Security, School of the Environment and Safety Engineering, Jiangsu University, Zhenjiang, Jiangsu 212013, China

[2] Jiangsu Collaborative Innovation Center of Technology and Material of Water Treatment, Suzhou University of Science and Technology, Suzhou 215009, China

[3] Beijing Key Laboratory of Functional Materials for Building Structure and Environment Remediation, School of Environment and Energy Engineering, Beijing University of Civil Engineering and Architecture, Beijing, 100044, China

* Correspondence: chenkun_kunkun@ujs.edu.cn (K.C.); wangchongchen@bucea.edu.cn (Chong-Chen Wang)

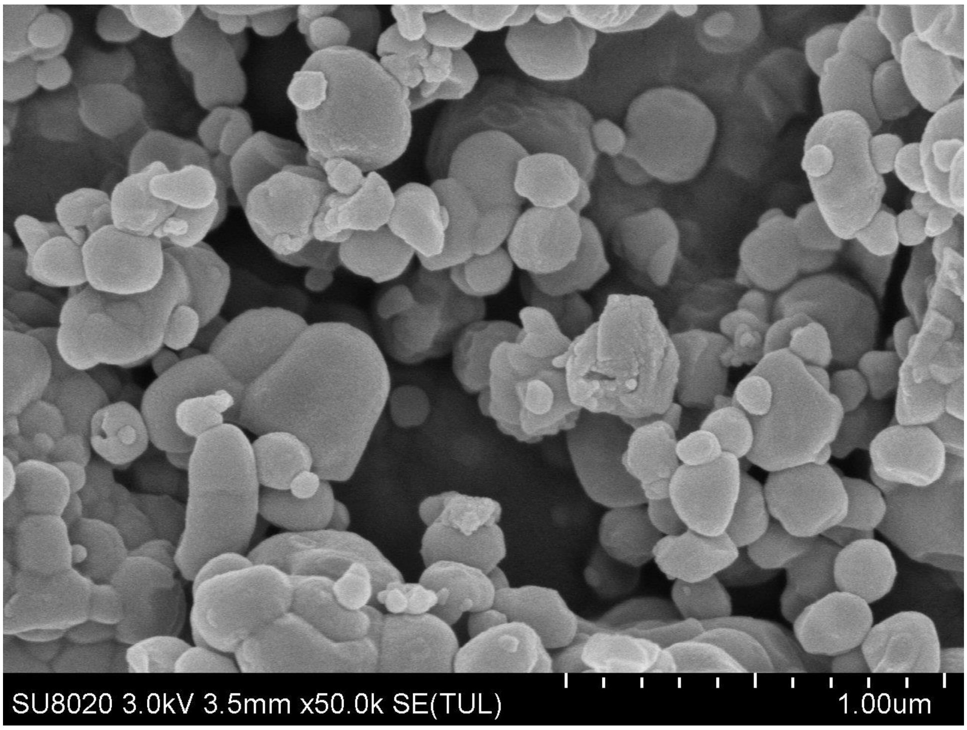

Figure S1. Scanning electron microscopy (SEM) of the zeolitic imidazolate framework-8 nanoparticles. The zeolitic imidazolate framework-8 nanoparticles (ZIF-NPs) were produced *via* the mechanochemical reaction between ZnO and 2-methylimidazole with a molar ratio of 1:2 (Taheri et al., 2020).

References:

Taheri, M., Bernardo, I.D., Lowe, A., Nisbet, D.R., Tsuzuki, T., 2020. Green full conversion of ZnO nanopowders to well-dispersed zeolitic imidazolate framework-8 (ZIF-8) nanopowders via a stoichiometric mechanochemical reaction for fast dye adsorption. Cryst. Growth Des. 20, 2761-2773. 10.1021/acs.cgd.0c00129.
